# Assessing the Protective Role of Epigallocatechin Gallate (EGCG) against Water-Pipe Smoke-Induced Toxicity: A Comparative Study on Gene Expression and Histopathology

**DOI:** 10.3390/molecules28227502

**Published:** 2023-11-09

**Authors:** Wajdy Al-Awaida, Khang Wen Goh, Hamzeh J. Al-Ameer, Yulia Sh. Gushchina, Vladimir I. Torshin, Alexandr E. Severin, Omar Al Bawareed, Besan Srour, Jude Al Farraj, Islam Hamad

**Affiliations:** 1 Department of Biology and Biotechnology, American University of Madaba, Madaba 11821, Jordan; b.srour@aum.edu.jo (B.S.); j.farraj@aum.edu.jo (J.A.F.); 2 Faculty of Data Science and Information Technology, INTI International University, Nilai 71800, Malaysia; khangwen.goh@newinti.edu.my; 3 Department of Biotechnology, Faculty of Allied Medical Sciences, Al-Ahliyya Amman University (AAU), Amman 19328, Jordan; hamzeh_uj@yahoo.com; 4 Department of General and Clinical Pharmacology, Medical Institute, Peoples’ Friendship University of Russia (RUDN University), 6 Miklukho-Maklaya Street, 117198 Moscow, Russia; gushchina-yush@rudn.ru; 5 Department of Normal Physiology, Medical Institute, Peoples’ Friendship University of Russia (RUDN University), 6 Miklukho-Maklaya Street, 117198 Moscow, Russia; torshin-vi@rudn.ru (V.I.T.); aesever@mail.ru (A.E.S.); alomar442@mail.ru (O.A.B.); 6 Department of Pharmacy, Faculty of Health Sciences, American University of Madaba, Madaba 11821, Jordan; i.hamad@aum.edu.jo

**Keywords:** epigallocatechin gallate (EGCG), water-pipe smoke, inflammatory markers, antioxidant genes, gene expression, oxidative stress, histopathological examination

## Abstract

Exposure to water-pipe smoking, whether flavored or unflavored, has been shown to instigate inflammation and oxidative stress in BALB/c mice. This consequently results in alterations in the expression of inflammatory markers and antioxidant genes. This study aimed to scrutinize the impact of Epigallocatechin gallate (EGCG)—a key active component of green tea—on inflammation and oxidative stress in BALB/c mice exposed to water-pipe smoke. The experimental setup included a control group, a flavored water-pipe smoke (FWP) group, an unflavored water-pipe smoke (UFWP) group, and EGCG-treated flavored and unflavored groups (FWP + EGCG and UFWP + EGCG). Expression levels of IL-6, IL1B, TNF-α, CAT, GPXI, MT−I, MT−II, SOD−I, SOD−II, and SOD-III were evaluated in lung, liver, and kidney tissues. Histopathological changes were also assessed. The findings revealed that the EGCG-treated groups manifested a significant decline in the expression of inflammatory markers and antioxidant genes compared to the FWP and UFWP groups. This insinuates that EGCG holds the capacity to alleviate the damaging effects of water-pipe smoke-induced inflammation and oxidative stress. Moreover, enhancements in histopathological features were observed in the EGCG-treated groups, signifying a protective effect against tissue damage induced by water-pipe smoking. These results underscore the potential of EGCG as a protective agent against the adverse effects of water-pipe smoking. By curbing inflammation and oxidative stress, EGCG may aid in the prevention or mitigation of smoking-associated diseases.

## 1. Introduction

Water-pipe smoking, also known as hookah or shisha, has experienced a surge in popularity globally, predominantly among the youth [1]. The once prevalent belief that water-pipe smoking, particularly with flavored tobacco, is less detrimental than traditional cigarettes is now being challenged by increasing scientific evidence [2,3]. Recent studies emphasize that both flavored and unflavoured water-pipe smoke (WPS) contain a plethora of harmful compounds, such as carcinogens and heavy metals, which can induce oxidative stress, inflammation, and potential organ damage [3,4]. There is also emerging evidence suggesting that flavored WPS might exhibit different toxicological properties than its unflavoured counterpart, a topic this study aims to delve deeper into [3].

Cellular defense against free radicals primarily hinges on a set of antioxidant enzymes, like superoxide dismutases (SODs), metallothioneins (MTs), catalase (CAT), and glutathione peroxidases (Gpxs). These enzymes act as a protective shield, preventing cellular damage from reactive oxygen species (ROS) [5,6]. Out of these, SODs are the frontline defense against the superoxide anion, with three distinct isoforms found in mammals, SOD−I, SOD−II, and SOD−III, each localized in specific cell compartments. SODs transform the superoxide anion into hydrogen peroxide (H_2_O_2_) and molecular oxygen (O2) [6,7]. Excessive levels of H_2_O_2_ can be cell-damaging [8]. To neutralize its harmful effects, the CAT enzyme, predominantly located in peroxisomes, catalyzes the breakdown of H_2_O_2_ into water and molecular oxygen, countering oxidative damage [9]. Without CAT in mitochondria, H_2_O_2_ reduction is managed by glutathione peroxidase 1 (GPX1) [10]. Additionally, MTs, cysteine-abundant proteins, encompass four variants (MT-1 to MT-4) in mammals, with MT-1 and MT-2 being ubiquitously distributed across tissues. Their functions are central in metal detoxification, oxidative stress response, and immune defense [11]. Notably, antioxidant metallothionein overexpression has been observed to shield against cardiac abnormalities instigated by nicotine exposure, mainly by curbing ROS accumulation and apoptosis [12].

Smoking, a primary contributor to many ailments, induces inflammatory reactions largely by releasing specific proteins or cytokines like TNFα, IL6, and IL1β [13]. Research has demonstrated that smoking upregulates these cytokines, accentuating inflammation [14]. In particular, IL1β and IL6 have critical roles in cell functions and immune responses, and their concentrations are markedly influenced by smoking [14,15]. Hence, analyzing IL1β, IL6, and TNFα gene expressions is essential for comprehending the relationship between smoking and inflammation. Gaining insight into these markers may facilitate the formulation of specific treatments for smoking-associated diseases.

Natural compounds, especially flavonoids, are renowned for their protective attributes against drug-induced adverse effects. Green tea (Camellia sinensis) has been extensively praised for its health-promoting properties, attributed to its abundant antioxidants and anti-inflammatory agents [16,17]. Green tea is distinguished by its high catechin concentration, a flavonoid subset. Catechins, potent phenolic and antioxidant compounds, constitute the main bioactive components of green tea, conferring numerous health benefits [18]. Foremost among these catechins is epigallocatechin gallate (EGCG), which forms over 80% of the green tea catechin content [19]. Its antioxidant prowess is deemed superior among naturally occurring compounds, adeptly quelling free radicals and mitigating oxidative stress, thus potentially averting tissue injuries and chronic ailments [20].

Consistent scientific evidence underscores EGCG’s protective potential against oxidative damage, especially in essential organs like the heart, brain, and kidneys [21]. The protective modalities of EGCG have been meticulously examined, revealing its capabilities in maintaining cell integrity, diminishing inflammation, and even thwarting carcinogenic pathways [22,23]. Moreover, EGCG has shown promise in enhancing metabolic health, aiding weight regulation, and exhibiting neuroprotective attributes [24,25].

This research endeavors to amalgamate molecular analysis with histopathological assessments, delineating the contrasting effects of flavored versus unflavored water-pipe smoking. By examining the influence of Epigallocatechin gallate (EGCG), the prominent active component in green tea, our study investigates its efficacy in moderating inflammation and oxidative stress. We meticulously measured the expression of inflammatory markers and antioxidant genes in the lung, liver, and kidney tissues of BALB/c mice subjected to water-pipe smoke. Our methodology incorporates a strategic experimental design with control and EGCG-treated cohorts, each exposed to both flavored and unflavored smoke variants.

The innovation of this study is anchored in its comparative analysis, which scrutinizes the protective efficacy of EGCG against the toxicological impacts of different types of water-pipe smoke. Through an intricate evaluation of gene expression and histopathological changes, we seek to unveil the nuanced protective role of EGCG. This investigation stands to contribute substantially to the field, as it may confirm EGCG’s potential as a mitigative agent against the detrimental effects of water-pipe smoking. The implications are profound; our findings aim to inform and enhance preventive and therapeutic strategies, thereby fostering advancements in public health policies targeted at water-pipe smoking-related complications.

## 2. Results

### 2.1. The Anti-Inflammatory Effects of Epigallocatechin gallate (EGCG) on Inflammatory Marker (IL-6, IL1B, and TNF-α) Expression in BALB/c mice Exposed to Flavored and Unflavored Water-Pipe Smoke

From the provided data, it is evident that the gene expression levels for various inflammatory markers (IL-6, IL1B, and TNF-α) were differentially impacted by the respective treatments.

**Figure 1:**
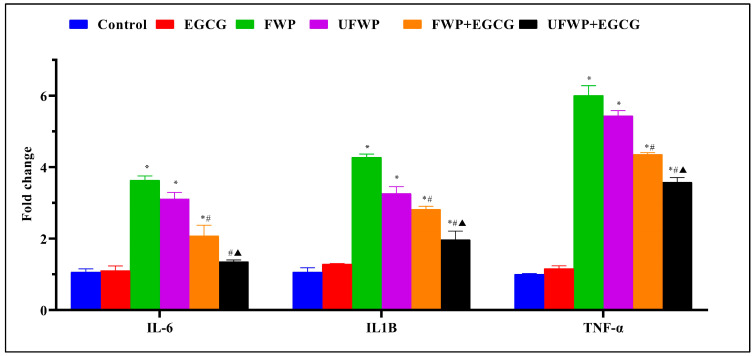
The Anti-Inflammatory Effects of Epigallocatechin Gallate (EGCG) on Inflammatory Markers IL-6, IL1B, and TNF-α in BALB/c mice Exposed to Flavored and Unflavored Water-Pipe Smoke. The figure represents the expression levels of inflammatory markers (IL-6, IL1B, and TNF-α) in control, FWP, UFWP, FWP + EGCG, and UFWP + EGCG groups. Exposure to both flavored and unflavored water-pipe smoke (FWP and UFWP groups) induced upregulation of these markers relative to the control group. However, EGCG treatment effectively reduced this overexpression in the FWP + EGCG and UFWP + EGCG groups, indicating its anti-inflammatory effects. Each value represents the mean ± standard deviation derived from multiple independent experiments. Statistical significance was set at *p* < 0.05. The keys (*, #, ▲) denote the specific significant differences between groups, *: Represents a significant difference between the control group and all other groups, #: Represents a significant difference between the FWP group and the FWP + EGCG group, ▲: Represents a significant difference between the UFWP group and the UFWP + EGCG group. The threshold for statistical significance was set at *p* < 0.05.

IL-6 levels significantly increased in the flavored water-pipe smoke (FWP) group (3.64 ± 0.12) and the unflavored water-pipe smoke (UFWP) group (3.11 ± 0.18) compared to the control group (1.07 ± 0.09). However, this significant increase is noticeably diminished upon EGCG treatment, as seen in the significantly decreased levels in the FWP + EGCG group (2.08 ± 0.30) and the UFWP + EGCG group (1.36 ± 0.05) (Figure 1, Table S1).

A similar pattern is observed for IL1B levels, with a significant increase in the FWP group (4.28 ± 0.09) and UFWP group (3.27 ± 0.19) relative to the control group (1.07 ± 0.12). Yet again, this significant escalation is mitigated with EGCG treatment, leading to significantly decreased levels in the FWP + EGCG (2.82 ± 0.08) and UFWP + EGCG (1.97 ± 0.24) groups (Figure 1, Table S1).

For TNF-α, the control group demonstrates a level of 1.01 ± 0.02. This level significantly increases in the FWP (6.01 ± 0.28) and UFWP (5.44 ± 0.15) groups. Consistent with the other markers, EGCG treatment results in a significant decrease in inflammation, as reflected in the reduced levels in the FWP + EGCG (4.36 ± 0.05) and UFWP + EGCG (3.58 ± 0.13) groups (Figure 1, Table S1).

In conclusion, the data indicate a potent anti-inflammatory effect of EGCG in BALB/c mice exposed to both flavored and unflavored water-pipe smoke. This is substantiated by the significantly decreased expression of the inflammatory markers IL-6, IL1B, and TNF-α (Figure 1, Table S1). The EGCG-treated group exhibited no significant difference in the expression of Inflammatory Markers IL-6, IL1B, and TNF-α compared to the control group.

### 2.2. Modulating Effects of Epigallocatechin gallate (EGCG) on Antioxidant Gene Expression in Kidney Tissues Exposed to Flavored and Unflavored Water-Pipe Smoke

The group treated with EGCG alone showed no marked variation in the expression of Antioxidant Genes CAT, GPXI, MT−I, MT−II, SOD−I, SOD−II, and SOD-III relative to the control group.

**Figure 2:**
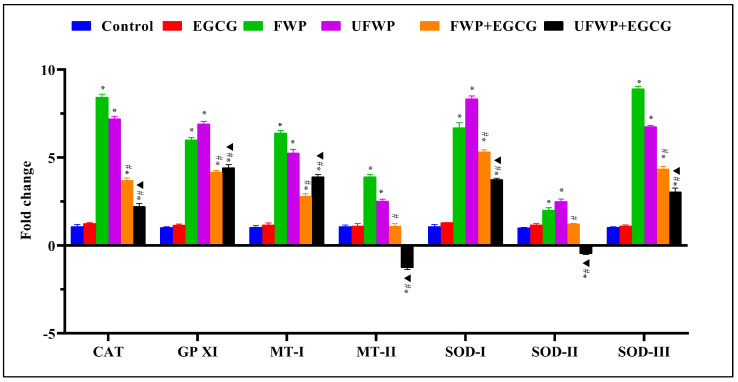
Modulating Effects of Epigallocatechin Gallate (EGCG) on Antioxidant Gene Expression in Kidney Tissues Exposed to Flavored and Unflavored Water-Pipe Smoke. The figure shows the relative expression of antioxidant genes (CAT, GPXI, MT-I, MT-II, SOD-I, SOD-II, SOD-III) in control, FWP, UFWP, FWP + EGCG, and UFWP + EGCG groups. For all genes examined, exposure to both flavored and unflavored water-pipe smoke (FWP and UFWP groups) led to an increased expression compared to the control. EGCG treatment effectively reduced these elevated levels in the FWP + EGCG and UFWP + EGCG groups, with MT-II and SOD-II in UFWP + EGCG even presenting with a decrease below control levels. Each value represents the mean ± standard deviation derived from multiple independent experiments. The keys (*, #, ▲) denote the specific significant differences between groups, *: Represents a significant difference between the control group and all other groups, #: Represents a significant difference between the FWP group and the FWP + EGCG group, ▲: Represents a significant difference between the UFWP group and the UFWP + EGCG group. The threshold for statistical significance was set at *p* < 0.05.

The expression of the Catalase (CAT) gene significantly increases in groups exposed to flavored water-pipe smoke (FWP, 8.43 ± 0.18) and unflavored water-pipe smoke (UFWP, 7.20 ± 0.14) compared to the control group (1.07 ± 0.12). However, EGCG treatment significantly decreases these elevated levels in both the FWP + EGCG (3.70 ± 0.14) and UFWP + EGCG (2.22 ± 0.17) groups (Figure 2, Table S2).

A similar pattern is evident for Glutathione Peroxidase XI (GPXI). The FWP (6.00 ± 0.14) and UFWP (6.91 ± 0.15) groups showed significant increases compared to the control group (1.02 ± 0.04). However, this significant rise is moderated upon EGCG treatment in the FWP + EGCG (4.17 ± 0.10) and UFWP + EGCG (4.42 ± 0.17) groups (Figure 2, Table S2).

Metallothionein-I (MT−I) gene expression follows the same trend, with significant increases in the FWP (6.40 ± 0.14) and UFWP (5.25 ± 0.21) groups compared to the control group (1.04 ± 0.09). EGCG treatment significantly lowers this spike in the FWP + EGCG (2.80 ± 0.14) and UFWP + EGCG (3.90 ± 0.13) groups (Figure 2, Table S2).

For the Metallothionein-II (MT−II) gene, expression significantly increased in the FWP (3.90 ± 0.14) and UFWP (2.53 ± 0.11) groups compared to the control group (1.07 ± 0.09). Notably, EGCG treatment significantly lowers this spike in the FWP + EGCG (1.09 ± 0.16 and UFWP + EGCG (−1.27 ± 0.10) groups (Figure 2, Table S2).

Superoxide Dismutase I (SOD−I), essential for reactive oxygen species detoxification, experiences a significant escalation in the FWP (6.70 ± 0.28) and UFWP (8.33 ± 0.17) groups compared to the control group (1.07 ± 0.12). EGCG treatment significantly decreases this rise in the FWP + EGCG (5.32 ± 0.11) and UFWP + EGCG (3.75 ± 0.07) groups (Figure 2, Table S2).

In contrast, Superoxide Dismutase II (SOD−II) reveals a milder but still significant increase in the FWP (2.00 ± 0.14) and UFWP (2.50 ± 0.14) groups. Interestingly, EGCG treatment significantly lowers this spike in the FWP + EGCG (1.23 ± 0.04) and UFWP + EGCG (−0.48 ± 0.04) groups.

Lastly, for Superoxide Dismutase III (SOD−III), EGCG treatment significantly decreases the enhanced expression induced by smoke, demonstrated by the significantly lower levels in the FWP + EGCG (4.35 ± 0.14) and UFWP + EGCG (3.05 ± 0.21) groups. This decline underscores the potency of EGCG in counteracting the oxidative stress caused by both flavored (8.91 ± 0.15) and unflavored (6.76 ± 0.08) water-pipe smoke (Figure 2, Table S1).

### 2.3. Modulating Effects of Epigallocatechin Gallate (EGCG) on Antioxidant Gene Expression in Liver Tissues Exposed to Flavored and Unflavored Water-Pipe Smoke

The group treated with EGCG alone showed no marked variation in the expression of Antioxidant Genes CAT, GPXI, MT−I, MT−II, SOD−I, SOD−II, and SOD−III relative to the control group.

**Figure 3:**
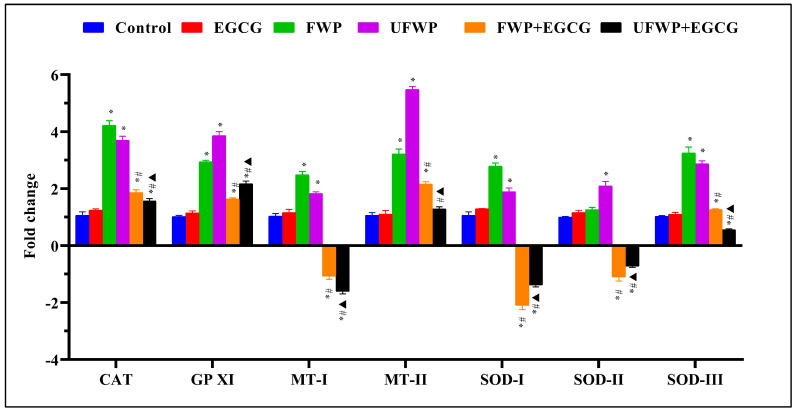
Modulating Effects of Epigallocatechin Gallate (EGCG) on Antioxidant Gene Expression in Liver Tissues Exposed to Flavored and Unflavored Water-Pipe Smoke. The figure illustrates the relative expression of antioxidant genes (CAT, GPXI, MT-I, MT-II, SOD-I, SOD-II, SOD-III) in control, FWP, UFWP, FWP + EGCG, and UFWP + EGCG groups. Animals in the FWP and UFWP groups exhibited an increase in the expression of all measured genes compared to the control, with CAT and GPXI levels significantly reduced upon EGCG treatment. Intriguingly, EGCG treatment resulted in MT-I, SOD-I, and SOD-II expression levels in the FWP + EGCG and UFWP + EGCG groups that were lower than the control group, indicating a potential inhibitory effect of EGCG on these genes’ expression upon smoke exposure. For MT-II and SOD-III, while EGCG treatment reduced expression levels, they remained above control in the FWP + EGCG group and decreased significantly in the UFWP + EGCG group. Each value is presented as the mean ± standard deviation derived from multiple independent experiments. The keys (*, #, ▲) denote the specific significant differences between groups, *: Represents a significant difference between the control group and all other groups, #: Represents a significant difference between the FWP group and the FWP + EGCG group, ▲: Represents a significant difference between the UFWP group and the UFWP + EGCG group. The threshold for statistical significance was set at *p* < 0.05.

For the Catalase (CAT) gene, the groups exposed to FWP (4.22 ± 0.17) and UFWP (3.70 ± 0.14) significantly increased in expression compared to the control group (1.07 ± 0.12). However, the elevated levels were significantly mitigated with EGCG treatment in both FWP + EGCG (1.86 ± 0.10) and UFWP + EGCG (1.57 ± 0.09) groups (Figure 3, Table S3).

A similar trend was observed for the Glutathione Peroxidase XI (GPXI) gene. Expression significantly increased in the FWP (2.94 ± 0.06) and UFWP (3.86 ± 0.14) groups compared to the control group (1.02 ± 0.04). Upon EGCG treatment, these heightened levels were significantly reduced in the FWP + EGCG (1.64 ± 0.05) and UFWP + EGCG (2.17 ± 0.10) groups (Figure 3, Table S3).

For the Metallothionein-I (MT−I) gene, expression significantly increased in the FWP (2.49 ± 0.12) and UFWP (1.83 ± 0.06) groups compared to the control group (1.04 ± 0.09). Intriguingly, the EGCG-treated groups, FWP + EGCG (−1.08 ± 0.11) and UFWP + EGCG (−1.61 ± 0.08) showed expression levels significantly lower than the control group, suggesting that EGCG might suppress this gene’s expression upon smoke exposure (Figure 3, Table S3).

The Metallothionein-II (MT−II) gene followed a similar pattern. Expression significantly increased in the FWP (3.22 ± 0.17) and UFWP (5.48 ± 0.11) groups compared to the control group (1.07 ± 0.09). With EGCG treatment, the levels were reduced but remained higher than the control in the FWP + EGCG (2.16 ± 0.08) group and significantly decreased in the UFWP + EGCG (1.29 ± 0.08) group (Figure 3, Table S3).

For Superoxide Dismutase I (SOD−I), the expression level significantly increased in the FWP (2.79 ± 0.11) and UFWP (1.90 ± 0.13) groups compared to the control group (1.07 ± 0.12). Surprisingly, the EGCG-treated groups, FWP + EGCG (−2.10 ± 0.14) and UFWP + EGCG (−1.39 ± 0.06) exhibited levels significantly lower than the control group (Figure 3, Table S3).

The Superoxide Dismutase II (SOD−II) gene exhibited a minor but still significant increase in the FWP (1.26 ± 0.08) and UFWP (2.10 ± 0.16) groups compared to the control group (1.01 ± 0.02). Surprisingly, the EGCG-treated groups, FWP + EGCG (−1.10 ± 0.14) and UFWP + EGCG (−0.73 ± 0.04) exhibited levels significantly lower than the control group (Figure 3, Table S3).

Lastly, for Superoxide Dismutase III (SOD−III), expression was significantly higher in the FWP (3.25 ± 0.21) and UFWP (2.88 ± 0.11) groups than in the control group (1.03 ± 0.04). After EGCG treatment, the expression significantly decreased but remained above control levels in FWP + EGCG (1.27 ± 0.04) and UFWP + EGCG (0.56 ± 0.03) groups (Figure 3, Table S3).

### 2.4. Modulating Effects of Epigallocatechin Gallate (EGCG) on Antioxidant Gene Expression in Lung Tissues Exposed to Flavored and Unflavored Water-Pipe Smoke

The group treated with EGCG alone showed no marked variation in the expression of Antioxidant Genes CAT, GPXI, MT−I, MT−II, SOD−I, SOD−II, and SOD-III relative to the control group.

**Figure 4:**
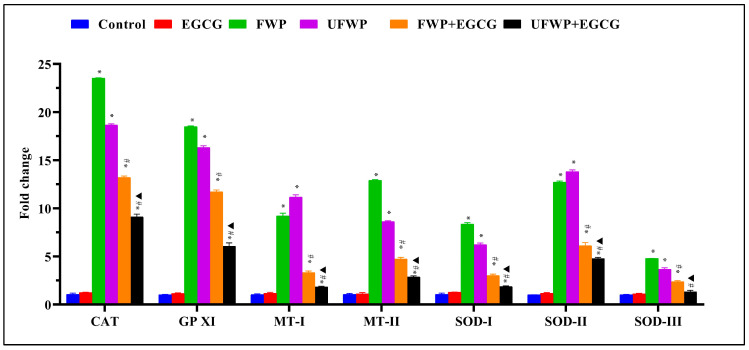
Modulating Effects of Epigallocatechin Gallate (EGCG) on Antioxidant Gene Expression in Lung Tissues Exposed to Flavored and Unflavored Water-Pipe Smoke. The figure shows the relative expression of antioxidant genes (CAT, GPXI, MT-I, MT-II, SOD-I, SOD-II, SOD-III) in control, FWP, UFWP, FWP + EGCG, and UFWP + EGCG groups. In the FWP and UFWP groups, exposure to water-pipe smoke led to a significant increase in the expression of all examined genes compared to control. EGCG treatment notably mitigated this increase across all genes, with both FWP + EGCG and UFWP + EGCG groups displaying significantly reduced expression levels compared to their non-EGCG treated counterparts. Each value represents the mean ± standard deviation derived from multiple independent experiments. The keys (*, #, ▲) denote the specific significant differences between groups, *: Represents a significant difference between the control group and all other groups, #: Represents a significant difference between the FWP group and the FWP + EGCG group, ▲: Represents a significant difference between the UFWP group and the UFWP + EGCG group. The threshold for statistical significance was set at *p* < 0.05.

The expression levels of the Catalase (CAT) gene in lung tissue significantly increased in the groups exposed to flavored (23.53 ± 0.04) and unflavored (18.66 ± 0.13) water-pipe smoke compared to the control group (1.07 ± 0.12). Notably, EGCG treatment significantly reduced these elevated levels in both the FWP + EGCG (13.21 ± 0.15) and UFWP + EGCG (9.11 ± 0.30) groups (Figure 4, Table S4).

A similar trend is observed in Glutathione Peroxidase XI (GPXI) expression, with significantly heightened levels in the FWP (18.50 ± 0.06) and UFWP (16.33 ± 0.18) groups relative to the control group (1.02 ± 0.04). This significant increase is notably attenuated by EGCG treatment, as seen in the FWP + EGCG (11.71 ± 0.20) and UFWP + EGCG (6.06 ± 0.36) groups (Figure 4, Table S4).

The Metallothionein-I (MT−I) gene follows this pattern, showing significantly elevated expression in the FWP (9.21 ± 0.30) and UFWP (11.17 ± 0.24) groups compared to the control group (1.04 ± 0.09). This significant increase is notably mitigated by EGCG treatment, with expression in the FWP + EGCG (3.32 ± 0.17) and UFWP + EGCG (1.84 ± 0.08) groups dropping considerably (Figure 4, Table S4).

Metallothionein-II (MT−II) expression also significantly increased in the FWP (12.91 ± 0.08) and UFWP (8.62 ± 0.08) groups from the control group level of 1.07 ± 0.09. The impact of EGCG treatment is significant, markedly reducing expression to 4.72 ± 0.18 (FWP + EGCG) and 2.88 ± 0.11 (UFWP + EGCG) (Figure 4, Table S4).

For Superoxide Dismutase I (SOD−I), we observe a significant upswing in expression levels in the FWP (8.36 ± 0.16) and UFWP (6.24 ± 0.16) groups relative to the control group (1.07 ± 0.12). Once again, EGCG treatment significantly mitigates this increase, lowering levels to 3.01 ± 0.16 (FWP + EGCG) and 1.86 ± 0.08 (UFWP + EGCG) (Figure 4, Table S4).

Superoxide Dismutase II (SOD−II) demonstrates a similar significant increase in the FWP (12.72 ± 0.12) and UFWP (13.82 ± 0.17) groups from a control level of 1.01 ± 0.02. With EGCG treatment, these expression levels are significantly reduced, as evident in the FWP + EGCG (6.12 ± 0.31) and UFWP + EGCG (4.79 ± 0.12) groups (Figure 4, Table S4).

Lastly, Superoxide Dismutase III (SOD−III) experiences a moderate but still significant increase in expression levels in the FWP (4.80 ± 0.01) and UFWP (3.67 ± 0.16) groups from the control level of 1.03 ± 0.04. Following EGCG treatment, these levels significantly decreased to 2.38 ± 0.11 (FWP + EGCG) and 1.32 ± 0.16 (UFWP + EGCG) (Figure 4, Table S4).

### 2.5. Histopathological Analysis

**Figure 5:**
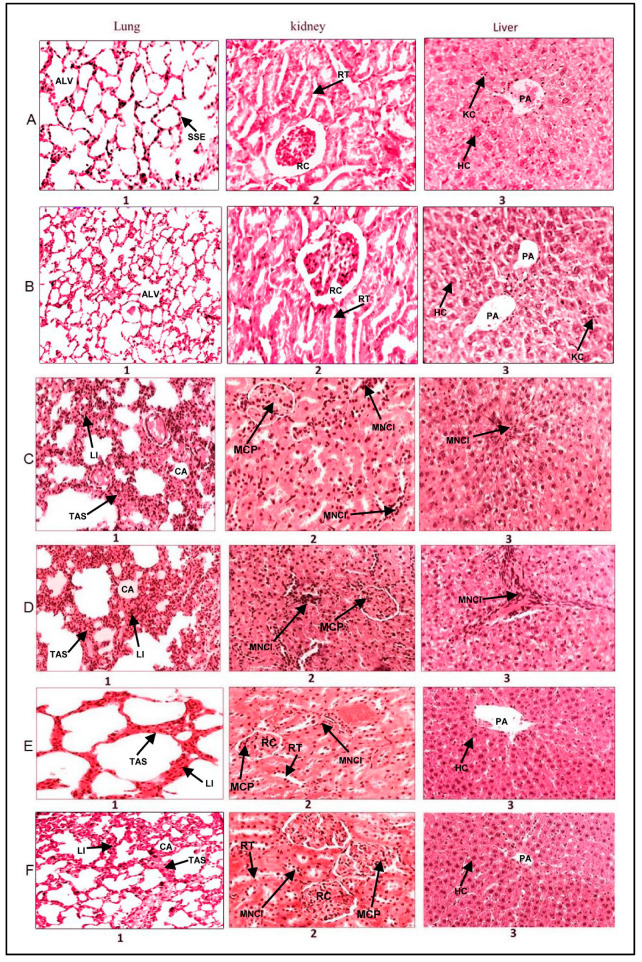
Effect of Epigallocatechin Gallate (EGCG) Consumption on the Morphology of Lung, Kidney, and Liver Tissues in Mice Exposed to Water-Pipe Smoke (Hematoxylin and Eosin Stain—H & E). Row (**A**): Displays typical morphology of the liver, lung, and kidney in a mouse exposed to clean air, which serves as the control. Row (**B**): Presents the liver, lung, and kidney morphology in an EGCG-treated mouse, demonstrating no observable pathological changes. Row (**C**): Reveals the morphology of liver, lung, and kidney tissues after exposure to unflavored water-pipe smoke. Specifically, C1, C2, and C3 highlight collapsed alveoli and inflammatory cell infiltration in the lungs, mesangial cell proliferation in kidneys, and inflammation in the liver’s portal area. Row (**D**): Illustrates the morphology of liver, lung, and kidney tissues in mice exposed to flavored water-pipe smoke. D1, D2, and D3 point to collapsed alveoli and inflammatory cell infiltration in the lungs, mesangial cell proliferation in kidneys, and inflammation in the liver’s portal area. Row (**E**): Shows the morphology of liver, lung, and kidney tissues in mice exposed to unflavored water-pipe smoke but treated with EGCG. E1, E2, and E3 depict lung tissues with diminished alveolar wall thickening, absence of inflammatory cell infiltration, liver tissues devoid of mesangial cell proliferation, and kidney tissues without portal area inflammation. Row (**F**): Demonstrates the morphology of liver, lung, and kidney tissues in mice exposed to flavored water-pipe smoke but treated with EGCG. F1, F2, and F3 portray lung tissues with diminished alveolar wall thickening, absence of inflammatory cell infiltration, liver tissues free from mesangial cell proliferation, and kidney tissues without portal area inflammation. Tissue Key: Kidney: RT—Renal Tubules, RC—Renal Corpuscle, MCP—Mesangial Cells Proliferation, MNCI—Mononuclear Leukocytes Infiltration. Liver: HC—Hepatocyte Cell, PA—Portal Area, KC—Kupffer Cells, MNCI—Mononuclear Leukocytes Infiltration. Lung: SSE—Simple Squamous Epithelium, Alv—Alveolar Sac, TAS—Thick Alveolar Septum, MNCI—Mononuclear Leukocytes Infiltration, LI—Lymphocyte Infiltration, CA—Collapsed Alveoli.

Exposure to both flavored and unflavored water-pipe smoke instigated observable inflammatory alterations in the liver, lung, and kidney tissues of BALB/c mice. These modifications included interstitial inflammation characterized by lymphocyte and plasma cell infiltration in the lungs (Figure 5C,D), portal area inflammation in the liver, and mesangial cell proliferation in renal corpuscles (Figure 5C,D). Intriguingly, the administration of EGCG effectively mitigated these inflammatory changes induced by smoking, as indicated by the nearly normalized tissue morphology observed (Figure 5E,F).

A distinct variation in the degree of inflammation was observed between the flavored and unflavored water-pipe smoke exposure groups. Specifically, the flavored water-pipe smoke exposure resulted in more pronounced inflammation in the liver, lung, and kidney tissues (Figure 5D) when compared to their unflavored counterparts (Figure 5C). This is noteworthy, considering the effectiveness of EGCG treatment in attenuating these pathological alterations, as no significant deviations in tissue morphology were detected in EGCG-treated mice (Figure 5B).

## 3. Discussion

The present investigation substantiates the evolving scientific literature underscoring the therapeutic potency of Epigallocatechin gallate (EGCG) in mitigating both the oxidative stress and inflammation triggered by inhaling both flavored and unflavored water-pipe smoke. This examination employed BALB/c mouse models to emulate the biological interactions of humans with such smoke. This claim draws on the foundational research that illustrates the powerful anti-inflammatory and antioxidant properties of EGCG, thus spotlighting its potential utility in managing smoke-induced diseases [26,27].

An extensive review of the existing literature indicates diverse no-observed adverse-effect levels (NOAELs) for EGCG in both rats and mice. Specifically, two 90-day oral toxicity studies of green tea extract (GTE) identified NOAELs of 500 mg/kg [28,29]. Furthermore, NOAELs of 764 mg/kg/day and 820 mg/kg/day have been documented [30]. Remarkably, in 28-day oral investigations, these thresholds increased to 2000 mg/kg [31] and 2500 mg/kg/day [32]. Additionally, evaluations on the potential hepatotoxicity of various green tea extracts, encompassing hot water, methanolic, and phenolic fractions, showed no acute hepatotoxic effects in doses from 500 mg/kg to 2500 mg/kg [33].

Although these results suggest a reasonable safety margin for EGCG in animal models, it is acknowledged that the NOAELs and the biological effects can be influenced by multiple factors. These include extraction techniques, source plant material, and accompanying polyphenols. These complexities emphasize the challenges in direct extrapolation to human contexts [34,35,36].

To further contextualize our study, the chosen dose of 50 mg/kg/day has been used in previous research to explore the effects of EGCG on different diseases in mice [34,35,36,37,38]. This collection of studies validates our methodological approach and provides a robust foundation for our presented results.

Through rigorous evaluation during this study, a pronounced surge in the expression of inflammatory markers, specifically Interleukin-6 (IL-6), Interleukin 1 beta (IL1B), and Tumor Necrosis Factor-alpha (TNF-α), was observed. The pathological role of these markers, pivotal in sparking numerous inflammatory diseases, has been thoroughly established in contemporary medical research [39]. Interestingly, a significant increase in both inflammatory and oxidative stress markers was noticeably evident in mice exposed to flavored water-pipe smoke. This observation reinforces the prevalent belief that flavored tobacco is inherently more harmful [3]. It also aligns with recent studies suggesting that flavorings might amplify the harshness of tobacco, thereby intensifying irritation and subsequent inflammatory responses [40].

Contrarily, upon introducing EGCG to the experimental setup, a remarkable decrease in the expression of these inflammatory markers was noted, thereby validating the existing literature highlighting the capability of polyphenolic compounds like EGCG to efficiently suppress inflammatory responses [40,41].

Our investigation further delved into the impact of EGCG on the expression of antioxidant genes within the kidney, liver, and lung tissues of mice subjected to both flavored and unflavored water-pipe smoke. In line with previous research, a substantial increase in the expression of antioxidant genes, including Catalase (CAT), Glutathione Peroxidase XI (GPXI), Metallothionein-I (MT−I), Metallothionein-II (MT−II), Superoxide Dismutase I (SOD−I), Superoxide Dismutase II (SOD−II), and Superoxide Dismutase III (SOD−III), was observed. This trend suggests that smoke inhalation triggers oxidative stress, which in turn leads to an upregulated antioxidant gene expression [6,42].

Interestingly, the administration of EGCG was associated with a discernable decrease in the expression of these antioxidant genes across all examined tissues, hinting at a potential reduction in oxidative stress. This observation aligns with a plethora of research emphasizing the antioxidant capabilities of EGCG [6,17,19].

Flavored and unflavored water-pipe smoking was observed to upregulate the expression levels of inflammatory and antioxidant genes—including IL-6, IL1B, TNF-α, CAT, GPXI, MT−I, MT−II, SOD−I, SOD−II, and SOD−III—in the lung, liver, and kidney tissues of the animal model. This upregulation, indicative of the body’s defensive response to oxidative stress caused by exposure to harmful substances in water-pipe smoke, emphasizes the body’s efforts to counteract potential cellular damage through a surge in antioxidant and inflammatory genes. However, when treated with EGCG, a prominent polyphenol in green tea recognized for its robust antioxidant capabilities, there was a pronounced decrease in these gene expressions. This suggests that EGCG may reduce the body’s urgency to amplify its antioxidants when combating oxidative stress. Such findings align well with the study by Al-Awaida et al., [6], which delineates the benefits of green tea intake on antioxidant- and inflammation-related gene expressions triggered by nicotine, a substance that similarly induces oxidative stress. In essence, the protective and modulatory capacity of EGCG is underscored by the observed decrease in gene expressions post-exposure, echoing its potential role in mitigating the adverse effects of oxidative stress and underscoring the consistent themes presented in the referenced scientific literature.

Moreover, it is crucial to consider the inherent biochemical properties of EGCG as an electron donor and a proficient scavenger of reactive oxygen species (ROS) like superoxide, peroxyl, hydroxyl, and peroxynitrite radicals. These properties lend further credence to EGCG’s prospective usefulness as an antioxidant therapeutic in conditions characterized by oxidative stress and inflammation. The radical scavenging qualities of EGCG are instrumental to its antioxidant capacity and seem to be significantly implicated in the protective effects observed in our study [43].

This distinct characteristic of EGCG could also play a critical role in tempering the inflammatory response. By scavenging reactive oxygen species, EGCG could potentially stunt the propagation of the inflammatory cascade incited by oxidative stress, hence mitigating the overexpression of pro-inflammatory markers [44]. These additional findings provide mechanistic insights into the protective role of EGCG against the detrimental outcomes of water-pipe smoke exposure.

In aggregate, our findings provide substantial support for the therapeutic promise of EGCG in the context of smoke-associated diseases. Nonetheless, it is crucial to emphasize that EGCG is not a panacea for the adverse effects of water-pipe smoking. The primary public health goal should remain focused on reducing or quitting tobacco use. Future research endeavors should aim to further unravel the underlying mechanisms of EGCG’s protective effects, determine optimal dosing regimens, and investigate the long-term impacts of its consumption. Such concerted efforts could lay the groundwork for more effective strategies to offset the adverse health effects associated with water-pipe smoke exposure.

In this investigation, we also scrutinized the protective efficacy of EGCG against the injurious effects of water-pipe smoke exposure in lung, kidney, and liver tissues. Our findings are in harmony with preceding research into EGCG’s antioxidant and anti-inflammatory properties and provide compelling evidence of its potential to alleviate the morphological alterations instigated by water-pipe smoke exposure [6,17,44].

Mice exposed to clean air in our control group displayed normal tissue morphology in the lung, kidney, and liver, reinforcing previous research findings [45]. However, mice exposed to either unflavored or flavored water-pipe smoke manifested distinct pathological changes, aligning with studies suggesting that water-pipe smoking contributes to various health complications, including those affecting the lungs, kidneys, and liver [1,45].

Notably, smoke-exposed mice that received EGCG treatment demonstrated protected tissue morphology. These observations further endorse prior research positing that the polyphenolic compounds in EGCG confer potent antioxidant and anti-inflammatory effects capable of reducing cellular damage from toxins [46,47].

Despite these encouraging findings, it is important to underscore that EGCG should not be considered a universal remedy for the deleterious effects of water-pipe smoking. Further research is needed to fully understand how EGCG exerts these protective effects, along with studies on the optimal dosage, duration, and potential long-term impacts of EGCG consumption [48].

Public health initiatives should continue to highlight the detrimental health effects of water-pipe smoking and the importance of reducing exposure. However, our findings underscore the potential of EGCG as a complementary protective measure against the harmful effects of exposure to such substances.

## 4. Materials and Methods

### 4.1. Experimental Design and Conditions

#### 4.1.1. Animal Model

The study utilized 48 male BALB/c mice, aged 9–12 weeks and weighing 25–30 g.

#### 4.1.2. Grouping and Treatment Regimen

We divided the mice into six distinct groups, each comprising eight mice. For 90 days, each group underwent unique daily treatments as follows:

1. Control Group: Subjected to ambient air without any external treatment.
2. Flavored Smoke Group: Exposed to flavored water-pipe smoke.
3. Unflavored Smoke Group: Exposed to unflavored water-pipe smoke.
4. Flavored Smoke + EGCG Group: Exposed to flavored smoke and administered Epigallocatechin gallate (EGCG; Sigma Aldrich, PHR1333) at a dosage of 50 mg/kg [36,37,38].
5. Unflavored Smoke + EGCG Group: Exposed to unflavored smoke and administered EGCG at a dosage of 50 mg/kg.
6. Ambient Air + EGCG Group: Subjected to ambient air and given EGCG at a dosage of 50 mg/kg.

For groups receiving EGCG, the compound was administered orally via gavage precisely one hour before daily smoke exposure, in accordance with the protocol referenced in [49].

#### 4.1.3. Housing Conditions

Mice were accommodated in a controlled environment with a temperature of 22 ± 1 °C, relative humidity between 65–70%, and a 12-h light/dark cycle [50]. For sample collection, euthanasia was executed using phenobarbital at 100 mg/kg [51].

### 4.2. Smoke Exposure Mechanism

#### 4.2.1. Device

A digital smoke delivery system was utilized, featuring an 8 mm thick Plexiglas inhalation chamber measuring 30 cm × 22.5 cm × 10.5 cm. The chamber capacity accommodated eight BALB/c mice simultaneously [49]. The device aimed to evaluate the effects of flavored and unflavored narghile tobacco smoke using a vacuum pump system. An electronic timer and valve system regulated the chamber’s airflow, ensuring balanced smoke and fresh air exposure, thus preventing oxygen deprivation [49].

#### 4.2.2. Exposure Protocol

The 90-s smoking procedure was divided into three distinct phases:

1. In the first 30 s, mice were exposed to either flavored or unflavored tobacco smoke.
2. In the subsequent 30 s, fresh air was introduced into the chamber to purge any remaining smoke.
3. The final 30 s allowed the mice a period for respiration, during which they inhaled the introduced fresh air.

This entire 90-s cycle was repeated 20 times back-to-back during each daily session, which was conducted seven days a week. Consequently, the total exposure time amounted to 30 min per group, each day. Following this regimen, tissues (including lung, liver, and kidney) were collected for genetic analysis and histopathological examination.

### 4.3. RNA Isolation and cDNA Synthesis

#### 4.3.1. Sample Preparation

For this, 50 mg of the frozen tissue was mixed with 2 mL of lysis buffer using the Promega RNA extraction Mini Kit [32].

#### 4.3.2. RNA Purification and cDNA Synthesis

Total RNA was extracted from tissues using Promega RNA extraction Mini Kit reagent [32], according to the manufacturer’s protocol and instructions for RNA purification and isolation. All RNA samples were stored at –80C until further used for complementary DNA (cDNA) synthesis.

One microgram of the RNA samples was reversely transcribed into cDNA using reverse transcription with Improm-II Reverse Transcriptase Promega (Madison, WI, USA), following the manufacturer’s protocol and instructions.

### 4.4. Quantitative PCR (qPCR) and Gene Expression Analysis

#### 4.4.1. System and Reagents

The CFX96™ Thermal Cycler Real-Time System (Bio-Rad Laboratories, Hercules, CA, USA) was utilized using SYBR Green Master Mix Promega (Madison, WI, USA). Each 20 µL reaction mixture contained 10 μL of SYBR Green Master Mix, 0.5 μL of forward primer (5 μM), 0.5 μL of reverse primers (5 μM), 1 μL of cDNA template, and 8 μL of RNase-free water.

#### 4.4.2. Normalization and Gene Targeting

Expression levels of specific genes in different tissue types were evaluated, along with the housekeeping gene Glyceraldehyde 3-phosphate dehydrogenase (GAPDH; as a reference gene). The reactions were performed in triplicate for consistency [33]. The relative expression of genes was calculated using the comparative CT method, a 2^^-ΔΔCt^ method [33]. Specific primers are detailed in Table 1.

### 4.5. Histopathological Analysis

#### 4.5.1. Sample Preparation

After the 90-day protocol, tissue samples from the lungs, liver, and kidneys were fixed in 10% buffered formaldehyde for a week.

#### 4.5.2. Staining and Imaging

Tissue sections of 5 μm were stained with hematoxylin and eosin (H&E). A Leica microscope with a 16-bit camera documented the samples [3].

### 4.6. Statistical Analysis

#### 4.6.1. Software Utilization

All statistical computations were executed using GraphPad Prism version 8.0 (GraphPad Software, La Jolla, CA, USA).

#### 4.6.2. Data Normality

The data derived from the study were assessed for normal distribution across all experimental groups, thereby allowing for the use of parametric tests in data comparison.

#### 4.6.3. Experimental Replicates

Data were amassed from three distinct trials, each comprising five replicates. Results are presented as mean ± standard deviation (SD).

#### 4.6.4. Statistical Tests

For multiple group comparisons, a one-way analysis of variance (ANOVA) was conducted. Tukey’s post hoc test was employed following ANOVA for multiple comparisons. Student’s t-test was utilized for pairwise comparisons.

#### 4.6.5. Significance Level

A *p*-value less than 0.05 was deemed indicative of statistical significance.

## 5. Conclusions

Our study provides strong evidence demonstrating the mitigating effects of Epigallocatechin gallate (EGCG), a prominent compound found in green tea, on the inflammatory and oxidative stress responses caused by both flavored and unflavored water-pipe smoke exposure in BALB/c mice. Histopathological assessments further substantiated EGCG’s beneficial impact, revealing its capacity to protect against smoke-induced tissue damage in the liver, lungs, and kidneys. However, the exact molecular mechanisms by which EGCG exerts these protective effects remain unclear and warrant further investigation. Employing techniques such as RNA-sequencing and proteomics in future studies may provide more comprehensive insights into these pathways.

## Figures and Tables

**Table 1:** Primer Sequences for Target Genes in Gene Expression Analysis.

Gene	Forward Primer	Reverse Primer	Reference
*IL1β*	AAGGATGACGACAAGCCAAC	CGCTGTGCTGATGTACCAGT	[49]
*IL6*	CTGCAAGAGACTTCCATCCAG	AGTGGTATAGACAGGTCTGTTGG	[50]
*TNFα*	GAGGCCAATAAAATCATCATCCC	CTTCCCATAGACTCTGAGTAGCG	[49]
*MT1*	GCTGTCCTCTAAGCGTCACC	AGGAGCAGCAGCTCTTCTTG	[51]
*MT2*	CAAACCGATCTCTCGTCGAT	AGGAGCAGCAGCTTTTCTTG	[51]
*SOD1*	GTGATTGGGATTGCGCAGTA	TGGTTTGAGGGTAGCAGATGAGT	[7]
*SOD2*	TTAACGCGCAGATCATGCA	GGTGGCGTTGAGATTGTTCA	[7]
*SOD3*	CATGCAATCTGCAGGGTACAA	AGAACCAAGCCGGTGATCTG	[7]
*GPX1*	GAAGAACTTGGGCCATTTGG	TCTCGCCTGGCTCCTGTTT	[7]
*CAT*	TGAGAAGCCTAAGAACGCAATTC	CCCTTCGCAGCCATGTG	[7]
*GAPDH*	AACGACCCCTTCATTGAC	TCCACGACATACTCAGCAC	[7]

## Data Availability

All data generated or analyzed during this study are encompassed in the submitted article.

## Supplementary Materials

The following supporting information can be downloaded at: https://www.mdpi.com/article/10.3390/molecules28227502/s1, Table S1: The Anti-Inflammatory Effects of Epigallocatechin gallate (EGCG) on Inflammatory Markers expression IL-6, IL1B, and TNF-α in BALB/c mice Exposed to Flavored and Unflavored Water-Pipe Smoke; Table S2: Modulating Effects of Epigallocatechin gallate (EGCG) on Antioxidant Gene Expression in Kidney Tissues Exposed to Flavored and Unflavored Water-Pipe Smoke; Table S3: Modulating Effects of Epigallocatechin gallate (EGCG) on Antioxidant Gene Expression in Liver Tissues Exposed to Flavored and Unflavored Water-Pipe Smoke; Table S4: Modulating Effects of Epigallocatechin gallate (EGCG) on Antioxidant Gene Expression in Lung Tissues Exposed to Flavored and Unflavored Water-Pipe Smoke.
