# Supplementary material for: Assessing the Protective Role of Epigallocatechin Gallate (EGCG) against Water-Pipe Smoke-Induced Toxicity: A Comparative Study on Gene Expression and Histopathology
Source: Molecules. 2023 Nov 9;28(22):7502. doi: 10.3390/molecules28227502 (PMC10673035; doi:10.3390/molecules28227502)
Supplement: Supplementary file 1 [file molecules-28-07502-s001.zip › molecules-2622299-supplementary.pdf]

Supplementary Table S1. The Anti-Inflammatory Effects of Epigallocatechin gallate (EGCG) on Inflammatory Markers expression IL-6, IL1B, and TNF-α in BALB/c mice Exposed to Flavored and Unflavored Water-Pipe Smoke.

	**FWP**	**UFWP**	**FWP+EGCG**	**UFWP+EGCG**
IL-6	+2.57±0.12	+2.04±0.18	+1.01±0.30	+0.29±0.05
IL1B	+3.21±0.09	+2.20±0.19	+1.75±0.08	+0.90±0.24
TNF-α	+5.00±0.28	+4.43±0.15	+3.35±0.05	+2.57±0.13

Gene expression values are denoted as Mean ± SD, adjusted by subtracting control values. Positive values indicate an increase in levels when compared to the control. Negative values indicate a decrease in levels when compared to the control.

Supplementary Table S2.Modulating Effects of Epigallocatechin gallate (EGCG) on Antioxidant Gene Expression in Kidney Tissues Exposed to Flavored and Unflavored Water-Pipe Smoke.

	**FWP**	**UFWP**	**FWP+EGCG**	**UFWP+EGCG**
CAT	+7.36±0.18	+6.13±0.14	+2.63±0.14	+1.15±0.17
GP XI	+4.98±0.14	+5.89±0.15	+3.15±0.10	+3.40±0.17
MT-I	+5.36±0.14	+4.21±0.21	+1.76±0.14	+2.86±0.13
MT-II	+2.83±0.14	+1.46±0.11	+0.02±0.16	-2.34±0.10
SOD-I	+5.63±0.28	+7.26±0.17	+4.25±0.11	+2.68±0.07
SOD-II	+0.99±0.14	+1.49±0.14	+0.22±0.04	-1.49±0.04
SOD-III	+7.88±0.15	+5.73±0.08	+3.32±0.14	+2.02±0.21

Gene expression values are denoted as Mean ± SD, adjusted by subtracting control values. Positive values indicate an increase in levels when compared to the control. Negative values indicate a decrease in levels when compared to the control.

Supplementary Table S3.Modulating Effects of Epigallocatechin gallate (EGCG) on Antioxidant Gene Expression in Liver Tissues Exposed to Flavored and Unflavored Water-Pipe Smoke.

	**FWP**	**UFWP**	**FWP+EGCG**	**UFWP+EGCG**
CAT	+3.15±0.17	+2.63±0.14	+0.79±0.10	+0.50±0.09
GP XI	+1.92±0.06	+2.84±0.14	+0.62±0.05	+1.15±0.10
MT-I	+1.45±0.12	+0.79±0.06	-2.12±0.11	-2.65±0.08
MT-II	+2.15±0.17	+4.41±0.11	+1.09±0.08	+0.22±0.08
SOD-I	+1.72±0.11	+0.83±0.13	-3.17±0.14	-2.46±0.06
SOD-II	+0.25±0.08	+1.09±0.16	-2.11±0.14	-1.73±0.04
SOD-III	+2.22±0.21	+1.85±0.11	+0.24±0.04	-0.47±0.03

Gene expression values are denoted as Mean ± SD, adjusted by subtracting control values. Positive values indicate an increase in levels when compared to the control. Negative values indicate a decrease in levels when compared to the control.

Supplementary Table S4.Modulating Effects of Epigallocatechin gallate (EGCG) on Antioxidant Gene Expression in Lung Tissues Exposed to Flavored and Unflavored Water-Pipe Smoke.

	**FWP**	**UFWP**	**FWP+EGCG**	**UFWP+EGCG**
CAT	+22.46±0.04	+17.59±0.13	+12.14±0.15	+8.04±0.30
GP XI	+17.48±0.06	+15.31±0.18	+10.69±0.20	+5.04±0.36
MT-I	+8.17±0.30	+10.13±0.24	+2.28±0.17	+0.80±0.08
MT-II	+11.84±0.08	+7.55±0.08	+3.65±0.18	+1.81±0.11
SOD-I	+7.29±0.16	+5.17±0.16	+1.94±0.16	+0.79±0.08
SOD-II	+11.71±0.12	+12.81±0.17	+5.11±0.31	+3.78±0.12
SOD-III	+3.77±0.01	+2.64±0.16	+1.35±0.11	+0.29±0.16

Gene expression values are denoted as Mean ± SD, adjusted by subtracting control values. Positive values indicate an increase in levels when compared to the control. Negative values indicate a decrease in levels when compared to the control.
